# Absolute and relative reliability of acute effects of aerobic exercise on executive function in seniors

**DOI:** 10.1186/s12877-017-0634-x

**Published:** 2017-10-25

**Authors:** Lars Donath, Sebastian Ludyga, Daniel Hammes, Anja Rossmeissl, Nadin Andergassen, Lukas Zahner, Oliver Faude

**Affiliations:** 1 Department of Sport, Exercise and Health, University of Basel, Birsstrasse 320B, 4052 Basel, Switzerland; 2 Institute of Exercise Training and Computer Science in Sport, German Sport University Cologne, Köln, Germany

**Keywords:** Reproducibility, Instrumented climbing hold, SEM, CoV, Piezoelectric force sensors, Bouldering, Performance

## Abstract

**Background:**

Aging is accompanied by a decline of executive function. Aerobic exercise training induces moderate improvements of cognitive domains (i.e., attention, processing, executive function, memory) in seniors. Most conclusive data are obtained from studies with dementia or cognitive impairment. Confident detection of exercise training effects requires adequate between-day reliability and low day-to-day variability obtained from acute studies, respectively. These absolute and relative reliability measures have not yet been examined for a single aerobic training session in seniors.

**Methods:**

Twenty-two healthy and physically active seniors (age: 69 ± 3 y, BMI: 24.8 ± 2.2, VO_2peak_: 32 ± 6 mL/kg/bodyweight) were enrolled in this randomized controlled cross-over study. A repeated between-day comparison [i.e., day 1 (habituation) vs. day 2 & day 2 vs. day 3] of executive function testing (Eriksen-Flanker-Test, Stroop-Color-Test, Digit-Span, Five-Point-Test) before and after aerobic cycling exercise at 70% of the heart rate reserve [0.7 × (HR_max_ – HR_rest_)] was conducted. Reliability measures were calculated for pre, post and change scores.

**Results:**

Large between-day differences between day 1 and 2 were found for reaction times (Flanker- and Stroop Color testing) and completed figures (Five-Point test) at pre and post testing (0.002 < *p* < 0.05, 0.16 < ɳ_p_^2^ < 0.38). These differences notably declined when comparing day 2 and 3. Absolute between days variability (CoV) dropped from 10 to 5% when comparing day 2 vs. day 3 instead of day 1 vs. day 2. Also ICC ranges increased from day 1 vs. day 2 (0.65 < ICC < 0.87) to day 2 vs. day 3 (0.40 < ICC < 0.93). Interestingly, reliability measures for pre-post change scores were low (0.02 < ICC < 0.71). These data did not improve when comparing day 2 with day 3. During inhibition tests, reaction times showed excellent reliability values compared to the poor to fair reliability of accuracy.

**Conclusion:**

Notable habituation to the whole testing procedure should be considered as it increased the reliability of different executive function tests. Change scores of executive function after acute aerobic exercise cannot be detected reliably. Large intra- and inter-individual of responses to acute aerobic exercise in seniors can be presumed.

## Background

One-third of the global and nearly half of the Western population will be aged >60 years at the end of the twenty-first century [1]. The process of biological aging, particularly in later adulthood, goes along with deteriorations of physical and cognitive function [2]. Cognitive function is a robust predictor of mortality in older people at population level [3] and executive control, seems to predict the functional status during daily life in seniors [4]. Executive functions refer to a family of top-down cognitive processes underlying the organization and control of goal-directed behaviour [5]. According to Miyake et al. [6] inhibitory control (control of attention, behaviour and emotions to override a dominant or pre-potent response), working memory (storage, manipulation and retrieval of information) and cognitive flexibility (ability to flexibly shift between mental sets) are considered its core components [6].

Previous reviews led to the “executive function hypothesis”, which suggests that regular physical activity and exercise selectively elicit benefits in this cognitive domain [7, 8]. Although benefits of exercise targeting cardiovascular fitness were also reported for attention and long-term memory in older adults [9], some meta-analytical findings show no evidence for improvements of cognitive performance after a period of aerobic training. Nonetheless, improvements in cognitive function following chronic exercise are considered clinically relevant as most findings from observational studies suggest that regular exercise can delay the onset of future dementia [10], possibly due to a promotion of cognitive reserves protecting cognitive function in spite of disease or damage [11]. Even low but regular PA levels were found to be positively associated with cognitive function (>100.000 participants across 20 nations) during aging [12].

In contrast to the heterogeneous findings obtained from longitudinal studies, acute bouts of aerobic exercise seem to transiently improve several dimensions of cognitive function, whereby immediate and delayed benefits were pronounced for executive function [13]. A recent meta-analysis revealed that moderate aerobic exercise elicits particularly beneficial effects for inhibitory control, working memory and task-switching in preadolescent children and older adults compared to other age groups [14]. These acute improvements of cognitive function were reported for time-dependent measures and appeared to be independent from the participant’s fitness level. Acute exercise-induced changes of cortical, vascular, hemodynamic and metabolic functions [15–17] have been discussed as underlying mechanisms for improved cognitive control. Although benefits elicited by a single exercise bout are considered to be transient, such cognitive improvements are still of high practical relevance. One major advantage of acute effects over chronic effects is that independent of the fitness level they can be elicited quickly [14]. Furthermore, older adults may use a single exercise session as a strategy to prepare for situations demanding high executive control. Whereas both acute and chronic facilitation of executive function by exercise gained notable attention, the variability and reliability of employed tests for assessing cognitive domain in older adults as well as the reliability of acute effects of exercise have long been disregarded.

Thus, examining changes of executive function following acute exercise adequate detection of meaningful change following these interventions [18]. Therefore, the identification of baseline and/or post-exercise variability of the respective cognitive testing parameters is needed. Otherwise, a reliable justification of detrimental or beneficial effects on cognitive performance is hindered. In this regard, the quantification of day-to-day reliability of a certain variable reflecting executive function needs to be discussed towards 1) chance, 2) system-immanent errors and 3) biological variability [19]. A resulting fundamental question is “how reliable is a particular assessment tool and how precisely and reproducible can acute exercise training effects on executive function be identified”. This is of particular importance as a certain amount of error is inherent when testing human beings and, thus, reliability can be considered as the amount of error or variability which is accepted for a particular test [20]. Hence, it is important to know whether an acute change of performance (e.g., executive function) can be attributed to the intervention rather than to its day-to-day variation [21]. Day-to-day variation can be due to the training setting, age-group, occurring fatigue, activity level, disease status [18]. In this regard, relative (e.g., intra-class correlation coefficients) and absolute variability estimates (e.g., standard error of measurement or coefficients of variation) need to be identified for baseline and post-exercise executive function in an acute setting of healthy seniors.

Against this background, the present study aimed at investigating day-to-day reliability of a variety of tests on executive function before and after acute aerobic exercise (i.e., Eriksen Flanker Test, Stroop Test, Digit Span, and Five Point Test) in a group of healthy seniors. Thereby, various absolute and relative reliability indices were collected within a three days repeated measures design in an acute exercise setting. We aimed at disentangling whether acute changes of executive function do vary in seniors. Beside acute effects of exercise on executive function this information is needed to estimate the likelihood of detectable change also in future training studies on exercise and executive function.

## Methods

### Participants and general design

**Fig. 1:**
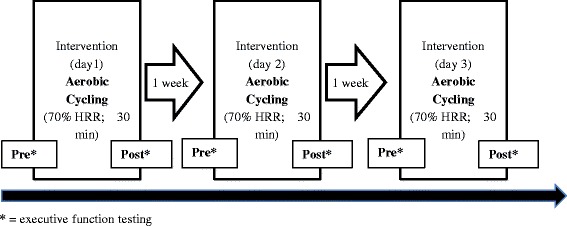
Study flow for the between day reliability study

Twenty-two healthy and physically and recreationally active seniors (Table 1) were recruited via a Senior Club (ProSenectute) and voluntarily participated in the present reliability study. Prior stroke, heart attack, heart failure and surgery, bypass, cardiac dysrhythmia, acute flu or cold, spinal-, joint and headpain, diabetes mellitus, untreated hypertension (>160/>100), acute and chronic inflammatory condition, severe arthrosis, recurrent vertigo, knee- or hipendoprosthesis, trauma within the last 6 month). None of the included participants reported any of those conditions. We conducted a repeated between-day comparison (i.e., day 1 vs. day 2 and day 2 vs. day 3) within weekly intervals (Fig. 1). None of the included participants reported any cardiac, pulmonary or neurological condition, elevated blood pressure or medication intake based on the physical activity readiness questionnaire (PAR-Q) [22]. Seniors with at least mild cognitive impairment (MCI) based on the Mini-mental state exam scoring between 20 and 25 [23] and clock drawing test [24] were excluded. None of the recruited seniors needed to be excluded due to at least mild MCI. The sample size of 20 Subjects was based on an at least moderate correlation between pre and post testing during executive function testing and deliver a strong power (1-beta error) of 90% and a very high alpha level of 0.01. We recruited more participants due to expected drop outs that did not occur.

**Table 1:** Anthropometric and performance-related data of the participants

	Men (*n* = 12) mean ± SD	Women (*n* = 10) mean ± SD	Total (*n* = 22) mean ± SD
Age (years)	68.3 ± 3.0	69.3 ± 3.8	68.8 ± 3.3
Height (cm)	176.0 ± 5.7	162.7 ± 5.5	170.0 ± 8.7
Weight (kg)	75.4 ± 5.4	66.9 ± 6.7	71.6 ± 7.3
BMI (kg/m^2)^	24.4 ± 2.2	25.3 ± 2.3	24.8 ± 2.2
Body fat (%)	21.8 ± 6.6	34.4 ± 4.4	27.5 ± 8.5
Resting heartrate (beats/min)	58.2 ± 7.7	64.9 ± 7.0	61.2 ± 8.0
Resting systolic blood pressure (mmHg)	143.4 ± 5.9	130.0 ± 18.9	137. 3 ± 14.8
Resting diastolic blood pressure (mmHg)	90.1 ± 7.7	77.3 ± 8.9	84.3 ± 10.3
Mini-mental status exam (MMSE)	28.8 ± 0.9	29.1 ± 1.1	29.0 ± 1.0
Clock drawing test	6.8 ± 0.6	6.6 ± 0.8	6.7 ± 0.7
Light physical activity (h/week)	10.3 ± 4.2	5.7 ± 6.2	8.2 ± 5.6
Sporting activities (h/week)	3.8 ± 5.3	3.4 ± 3.1	3.6 ± 4.3
Maximal heart rate (1/min)	159.1 ± 12.2	159.0 ± 10.7	159.0 ± 11.2
VO_2_peak (ml/min/kg)	36.0 ± 3.9	26.9 ± 2.3	31.9 ± 5.6
Maximal performance (Watts)	199.5 ± 29.0	118.6 ± 22.4	162.7 ± 48.5
Duration of Exercise testing (min)	14.5 ± 1.9	11.9 ± 2.5	13.3 ± 2.5
Respiratory exchange ratio (RER)	1.1 ± 0.0	1.1 ± 0.1	1.1 ± 0.0

All seniors were requested to refrain from moderate to severe exercise within the last 24 h prior to spiroergometric exercise testing or acute aerobic exercise training. Caffeine intake was not allowed 5 h prior to exercise testing or training. No caffeine withdrawal symptoms were observed. Between-day variability of cognitive function before and after acute aerobic exercise was assessed on three days in weekly intervals (Fig. 1). The study was approved by the local ethical committee of the University of Basel (11/23/2015–254) and meets the criteria of the declaration of Helsinki. After receiving all relevant study information, the participants signed an informed consent to the study including a permission to publish the data. The Freiburg physical activity questionnaire was used to assess baseline physical activity in h/week (Frey et al. 1995). The total amount of weekly physical activity includes baseline physical activity (e.g., daily walked or biked distance, stair climbing), leisure time activity (e.g., hiking, dancing, bowling) and sportive activity (disciplines). The summarized weekly hours were used to describe activity of both groups. Participants’ body fat and weight was assessed using the InBody 170 (JP Global Markets GmbH, Germany). To measure body height a measuring pole was used. These are common, valid and good to excellently reliable tools for anthropometric assessment (0.8 > *r* > 0.95).

### Aerobic cycling exercise and exercise intensity determination

Based on health-related exercise recommendations of the American College of Sports Medicine [25], 30 min of aerobic cycling exercise at 70% of the heart rate reserve (HRR) using the “Karvonen-formula” (0.7 × (HR_max_ – HR_rest_)) was applied in-between cognitive testing [26, 27]. In order to calculate HRR, maximal heart rates (HR_max_) were obtained from maximal spiroergometric ramp-like exercise testing on a treadmill. Briefly, the protocol started at a velocity of 6 km/h − 1 and an inclination of 0.1% for a time period of 1 min. Intensity was increased by 1 km/h − 1 every minute until volitional exhaustion was reached. Maximal exertion levels have been verified if at least three out of the following 5 criteria were reached: 1) age-predicted maximal heart rates [28], 2) breathing frequency (>35/min) 3) capillary lactate concentration (>8 mmol/L), 4) ventilator equivalent for oxygen uptake (>35) and 5) respiratory exchange ratio (RER >1.1) [29]. Ventilatory parameters were collected using the Cortex Metalyzer® 3B metabolic test system (Cortex Biophysik GmbH, Leipzig, Germany). VO_2peak_ and HR_max_ were derived from the final 30 s before exercise cessation.

### Cognitive testing

Different aspects of executive function were assessed using the Five-Point-Test [30] and computer-based modified versions of the Eriksen-Flanker task (Eriksen & Eriksen, 1974), Stroop Color-Word [31] as well as Digit-Span (forward & backward) in a counterbalanced order. All computer-based tests were administered by the same rater with Presentation 18.0 (NeuroBehavioral Systems, USA). No breaks between testing were allowed. Cognitive assessments were performed in a separate room with one participant at a time. Prior to testing, instructions were provided verbally in a standardized manner. Afterwards, instructions were also presented on the screen to make sure the participants understood the task. Following the instruction, noise was kept to a minimum. Environmental temperature was held constant at 21 °C during cognitive testing.

#### Flanker task

The modified Flanker task was used to assess the inhibitory component of executive control [32]. During the task, participants are required to respond to a centrally presented target stimulus (vertical visual angle: 8.5°) by pressing a button corresponding to its direction. In congruent trials, the target stimulus was surrounded by six arrows facing the same direction, whereas in incongruent trials the centrally presented target stimulus was facing in the opposite direction of the flanking arrows. Participants completed one practice block with 10 trials and one test block with 100 trials. In each trial, arrows were presented focally for 200 ms after a fixation period of 1000 ms. The response window was set to 1000 ms and participants received feedback on their response. Congruent and incongruent trials were presented in random order and with equal probability. Reaction time and accuracy for congruent and incongruent trials were calculated to assess speed processing and interference control.

#### Stroop color test (SCT)

The Stroop Color-Word is a standard test to assess inhibitory control [32, 33]. The stimulus used in this task is a color name presented in the centre of the screen (vertical visual angle: 8.5°). It is either printed in ink matching the color name (compatible trials) or in a different color of ink (incompatible trials). Participants are instructed to press a button corresponding to the ink in the color block or the name of the color in the word block. The colors/words chosen for this task were “rot” (red), “grün” (green), “gelb” (yellow) and “blau” (blue). Participants completed a practice block with 12 trials as well as one color and one word block with 96 trials each. The order of test blocks was counter-balanced across participants and both types of trials (compatible, incompatible) appeared with equal probability. Each trial started with a 500 ms fixation period, followed by the presentation of a stimulus over 200 ms. Responses were collected within a 2500 ms window and participants received feedback on their accuracy. As dependent measures of information processing and interference control reaction time and accuracy were calculated for compatible and incompatible trials, respectively.

#### Digit span forward and backward (DSF/DSB)

Digit Span Forward and backward is used to assess working memory and updating of working memory, respectively [32]. In this task, participants were required to repeat a sequence of digits (1–9 presented with equal probability) on a computer keyboard in the same (forward) and in reversed order (backward). Digits did not occur in regular ascending or descending sequences with equal consecutive step sizes. In all trials, each digit was presented for 500 ms with an inter-stimulus interval of 500 ms. Although participants were instructed to provide a timely response, the time window was not limited. The span length was increased by one digit every two trials (starting from 3 in digit span forward and 2 in backward), until the limit of two successive errors was reached. Measures obtained from the task were lengths of the longest span answered correctly forward and backward as well as the number of cumulative errors.

#### Five-point test

The Five-Point test is used for the assessment of figural fluency functions, which relate to the set-shifting component of executive control [33]. For this task, participants received a sheet of paper, on which 40 five-dot matrices were printed. Each matrix was identical and consisted of a fixed pattern of five symmetrically arranged dots. Participants were required to draw as many designs as possible in 2 min by connecting the dots with at least one straight line. After the investigator demonstrated two possible designs, participants were asked to perform the task. The Five-Point test was scored by counting the total number of unique designs and repetitions of designs (perseverative errors).

### Statistics

All outcome measures were checked for normal distribution (Kolmogorov Smirnov test) and variance homogeneity (Levene test). Data are given as means with standard deviations (SD) and 90% confidence intervals (90% CI), respectively.

Repeated measures analyses of variance were applied separately for each outcome measure between the two subsequent trials at the beginning and at the end of the testing procedure. An α-level of *p* < 0.05 was accepted as statistically significant. Effect sizes for variance analyses were given as partial eta squared (η_p_^2^) with values ≥0.01, ≥ 0.06, ≥ 0.14 indicating small, moderate, or large effects, respectively. Intraclass correlation coefficients as a measure of relative reliability was computed according to the formula ICC = 1 – (SEM^2^/SD^2^) with SD serving as between subject standard deviation [21].

We calculated the standard error of measurements (SEM, computed as the SD of the difference divided by the square root of 2) as well as the log-transformed coefficient of variation (CoV) together with 90% confidence limits as measures of absolute reliability [18, 19]. Reliability data were analysed using a published spreadsheet [34] in Microsoft Excel® of Hopkins (Hopkins 2007).

## Results

### Day-to-day variability between the 1st and 2nd day at pre and post testing

At pre testing, meaningful differences were observed between 1st and 2nd day of testing for the Eriksen-Flanker- (compatible: *p* = 0.05, ɳ_p_^2^ = 0.12) and Five-Point-test (correctly completed figures: p = 0.05, ɳ_p_^2^ = 0.20). The Stroop Color-Word test revealed relevant differences between 1st and 2nd day for compatible and incompatible reaction time at both pre- and post-testing (pre: 0.002 < *p* < 0.04, 0.16 < ɳ_p_^2^ < 0.38) (Table 2, column 4 and 5) Coefficients of variation below 10% were found for reaction times and accuracy on the Eriksen-Flanker- (0.5% < CoV < 5.5%) and Stroop-Color-test (0.8% < CoV < 8.7%), (Table 2, column 9). Digit-span testing at pre and post revealed very large CoV ranging between 15 and 43%, particularly for cumulative errors both backward and forward. ICC values were mainly good to excellent for the Eriksen-Flanker- (0.66 < ICC < 0.84), Five-Point-test (0.71 < ICC < 0.75) and Stroop Color-Word test (0.65% < ICC < 0.87, except for: accuracy, compatible pre: ICC: 0.28 and incompatible post: ICC: 0.25).

**Table 2:** Absolute and relative reliability data of various outcomes obtained from different executive function tests before and after acute aerobic cycling exercise for day 1 and 2 comparison

		INT day 1 mean (SD)	INT day 2 mean (SD)	rANOVA (*p*-value)	Effect size (ɳp^2^)	change in mean [90% CI]	SEM (Typical error) [90% CI]	CoV [90% CI]	ICC [90% CI]
Eriksen Flanker Test									
reaction time, congruent [ms]	pre	481 (67)	468.6 (53.6)	0.05	0.12	−9.1 [−24.2; 5.9]	27.6 [21.89; 37.78]	5.5 [4.4; 7.7]	0.81 [0.63; 0.90]
	post	461 (48)	451.2 (54.1)	0.48	0.03	−9.8 [−21.7; 2.0]	21.7 [17.22; 29.73]	4.8 [3.8; 6.6]	0.83 [0.67; 0.92]
accuracy, congruent [% correct]	pre	98.5 (3.3)	99.1 (2.4)	0.45	0.03	0.0 [−0.7; 0.7]	1.2 [1.0; 1.7]	1.3 [1.0; 1.7]	0.84 [0.68; 0.92]
	post	99.3 (1.2)	99.7 (0.9)	0.76	0.01	0.3 [0.0; 0.7]	0.5 [0.4; 0.7]	0.5 [0.4; 0.7]	0.77 [0.57; 0.88]
reaction time, incongruent [ms]	pre	545 (7)	536.7 (51.3)	0.38	0.04	−1.8 [−18.7; 15.2]	31.0 [24.6; 42.5]	5.3 [4.2; 7.4]	0.78 [0.58; 0.89]
	post	522 (59)	514.5 (56.5)	0.31	0.05	−6.3 [−20.5; 7.9]	26.0 [20.6; 35.6]	4.9 [3.9; 6.8]	0.81 [0.63;0.90]
accuracy, incongruent [% correct]	pre	94.5 (7.8)	96.5 (4.8)	0.19	0.08	1.6 [−0.2; 3.4]	3.2 [2.6; 4.4]	3.9 [3.1; 5.4]	0.77 [0.56; 0.88]
	post	96.9 (4.4)	96.9 (3.5)	0.68	0.02	0.0 [−1.3; 1.3]	2.4 [1.9; 3.3]	2.6 [2.1; 3.6]	0.66 [0.39; 0.82]
Stroop Color Test									
reaction time, compatible [ms]	pre	833 (105)	775 (95)	0.002	0.38	−51 [−74; −28]	42 [33; 58]	5.0 [3.9; 6.9]	0.84 [0.68; 0.92]
	post	781 (89)	744 (90)	0.04	0.16	−40 [−58; −22]	33 [26; 46]	4.4 [3.5; 6.1]	0.87 [0.75; 0.94]
accuracy, compatible [% correct]	pre	98.7 (1.4)	98.9 (1.4)	0.30	0.02	0.3 [−0.4; 0.9]	1.2 [1.0; 1.6]	1.2 [1.0; 1.7]	0.28 [−0.12; 0.58]
	post	98.3 (1.7)	98.8 (1.6)	0.10	0.08	0.4 [0.0; 0.8]	0.8 [0.6; 1.1]	0.8 [0.6; 1.1]	0.80 [0.61; 0.90]
reaction time, incompatible [ms]	pre	956 (134)	889 (114)	0.01	0.25	−69 [−11; −24]	82 [65; 112]	8.7 [6.8; 12.0]	0.59 [0.28; 0.78]
	post	902 (103)	882.1 (105)	0.02	0.17	−20 [−55; 15]	63 [50; 87]	6.9 [5.4; 9.5]	0.65 [0.37; 0.81]
accuracy, incompatible [% correct]	pre	97.3 (3.9)	97.8 (2.8)	0.32	0.06	0.6 [−0.4; 1.6]	1.8 [1.4; 2.5]	1.9 [1.5; 2.6]	0.73 [0.50; 0.86]
	post	98.0 (2.1)	97.7 (3.2)	0.52	0.02	−0.4 [−1.7; 0.9]	2.3 [1.9; 3.2]	2.5 [2.0; 3.4]	0.25 [−0.15; 0.56]
Digit Span									
forward [completed stages]	pre	6.3 (1.5)	6.1 (1.2)	0.21	0.03	−0.1 [−0.5; 0.4]	0.8 [0.7; 1.2]	14.7 [11.5; 20.6]	0.63 [0.33; 0.80]
	post	6.3 (1.5)	6.2 (1.0)	0.74	0.01	−0.1 [−0.5; 0.4]	0.9 [0.7; 1.2]	16.8 [13.1; 23.7]	0.50 [0.15; 0.72]
forward [cumulative errors]	pre	3.3 (1.0)	3.0 (1.0)	0.55	0.02	−0.2 [−0.8; 0.4]	1.0 [0.8; 1.4]	36.6 [28.1; 53.3]	−0.07 [−0.47; 0.31]
	post	3.0 (0.9)	3.0 (0.8)	0.81	0.01	0.1 [−0.5; 0.6]	1.0 [0.8; 1.4]	39.9 [30.5; 58.4]	−0.25 [−0.62; 0.14]
backward [completed stages]	pre	5.3 (2.0)	5.6 (1.3)	0.34	0.02	0.3 [−0.5; 1.0]	1.3 [1.1; 1.8]	43.3 [33.1; 63.8]	0.38 [0.00; 0.65]
	post	5.4 (1.3)	5.7 (1.5)	0.35	0.03	0.2 [−0.3; 0.7]	0.9 [0.7; 1.2]	17.7 [13.8; 25.1]	0.63 [0.34; 0.81]
backward [cumulative errors]	pre	3.3 (1.2)	2.9 (0.8)	0.20	0.05	−0.4 [−1.0; 0.2]	1.2 [0.9; 1.6]	43.3 [33.1; 63.8]	−0.28 [−0.64; 0.12]
	post	2.9 (0.8)	3.0 (0.7)	0.60	0.01	0.1 [−0.3; 0.5]	0.8 [0.6; 1.0]	30.2 [23.3; 43.5]	−0.01 [−0.42; 0.36]
Five-Point-Test									
correctly completed figures [n]	pre	24.8 (6.4)	28.7 (5.3)	0.05	0.20	3.2 [1.4; 5.0]	3.2 [2.6; 4.4]	14.7 [11.5; 20.7]	0.71 [0.47; 0.85]
	post	28.7 (5.0)	30.2 (4.4)	0.07	0.09	1.6 [0.2; 2.9]	2.4 [1.9; 3.3]	9.4 [7.4; 13.0]	0.75 [0.53; 0.87]
		INT day 2 mean (SD)	INT day 3 mean (SD)	rANOVA (*p*-value)	Effect size (ɳp^2^)	change in mean [90% CI]	SEM (Typical error) [90% CI]	CoV [90% CI]	ICC [90% CI]
Eriksen Flanker Test									
reaction time, congruent [ms]	pre	469 (54)	478 (78)	0.41	0.03	−1 [−14; 12]	23 [19; 32]	5.1 [4.0; 7.0]	0.89 [0.77; 0.94]
	post	451 (54)	454 (45)	0.68	0.01	4 [−6; 15]	19 [15; 26]	4.2 [3.3; 5.9]	0.86 [0.73; 0.93]
accuracy, congruent [% correct]	pre	99.1 (2.4)	99.3 (1.2)	0.74	0.01	0.3 [−0.7; 1.3]	1.9 [1.5; 2.5]	1.9 [1.5; 2.7]	0.07 [−0.34; 0.42]
	post	99.1 (09)	97.5 (10.6)	0.33	0.05	−2.5 [−6.9; 1.9]	8.0 [6.3; 10.9]	11.6 [9.1; 16.3]	−0.12 [−0.51; 0.27]
reaction time, incongruent [ms]	pre	537 (51)	520 (46)	0.02	0.19	−16 [−27; −6]	20 [15; 27]	3.9 [3.1; 5.4]	0.85 [0.71; 0.93]
	post	514 (56)	510 (55)	0.22	0.07	−6 [−14; 3]	15 [12; 21]	3.0 [2.3; 4.1]	0.93 [0.86; 0.97]
accuracy, incongruent [% correct]	pre	96.5 (4.8)	98.0 (3.9)	0.16	0.09	1.5 [−0.4; 3.4]	3.4 [2.7; 4.7]	3.9 [3.1; 5.3]	0.04 [0.04; 0.67]
	post	96.9 (3.5)	96.0 (10.5)	0.72	0.01	−1.3 [−6.0; 3.4]	8.5 [6.8; 11.7]	12.2 [9.5; 17.1]	−0.19 [−0.58; 0.20]
Stroop Color Test									
reaction time, compatible [ms]	pre	775 (95)	759 (81)	0.20	0.08	−23 [−44; −3]	37 [29; 51]	4.6 [3.7; 6.4]	0.84 [0.68; 0.92]
	post	744 (91)	730 (86)	0.16	0.09	−13 [−31; 4]	32 [25; 44]	4.3 [3.4; 6.0]	0.88 [0.76; 0.94]
accuracy, compatible [% correct]	pre	98.9 (1.4)	98.1 (1.7)	0.03	0.22	−0.7 [−1.2; −0.1]	1.0 [0.8; 1.4]	1.1 [0.8; 1.4]	0.59 [0.28; 0.78]
	post	98.8 (1.6)	99.3 (0.8)	0.05	0.17	0.5 [0.1; 1.0]	0.8 [0.6; 1.1]	0.8 [0.7; 1.2]	0.64 [0.35; 0.81]
reaction time, incompatible [ms]	pre	888 (114)	868 (103)	0.27	0.06	−17 [−47.6; 14.1]	56.4 [44.8; 77.3]	6.4 [5.0; 8.8]	0.75 [0.53; 0.87]
	post	882 (105)	870 (107)	0.47	0.03	−20 [−47; 7]	50 [39; 67]	5.6 [4.4; 7.7]	0.80 [0.61; 0.90]
accuracy, incompatible [% correct]	pre	97.8 (2.8)	98.4 (1.9)	0.23	0.07	0.7 [−0.3; 1.6]	1.7 [1.4; 2.4]	1.8 [1.5; 2.5]	0.49 [0.14; 0.72]
	post	97.7 (3.2)	98.1 (1.3)	0.54	0.02	0.5 [−0.7; 1.6]	2.1 [1.6; 2.8]	2.2 [1.7; 3.0]	0.29 [−0.11; 0.58]
Digit Span									
forward [completed stages]	pre	6.1 (1.2)	6.3 (0.95)	0.35	0.04	0.3 [−0.1; 0.7]	0.8 [0.6; 1.1]	13.4 [10.5; 18.8]	0.47 [0.11; 0.70]
	post	6.2 (1.0)	6.1 (1.2)	0.82	0.00	0.1 [−0.3; 0.4]	0.6 [0.5; 0.9]	10.8 [8.5; 15.0]	0.67 [0.40; 0.83]
forward [cumulative errors]	pre	3.0 (1.0)	3.1 (1.0)	0.63	0.01	0.2 [−0.4; 0.7]	1.0 [0.8; 1.3]	38.3 [29.3; 55.9]	0.05 [−0.36; 0.41]
	post	3.0 (0.8)	2.9 (0.8)	0.53	0.02	0.0 [−0.4; 0.4]	0.7 [0.5; 0.9]	24.0 [18.6; 34.2]	0.40 [0.02; 0.66]
backward [completed stages]	pre	5.6 (1.3)	5.5 (1.6)	0.70	0.01	−0.2 [−0.6; 0.3]	0.8 [0.6; 1.1]	15.7 [12.3; 22.2]	0.71 [0.47; 0.85]
	post	5.7 (1.5)	6.0 (1.6)	0.35	0.04	0.4 [−0.1; 0.8]	0.8 [0.6; 1.1]	15.7 [12.3; 22.1]	0.76 [0.55; 0.88]
backward [cumulative errors]	pre	2.9 (0.8)	3.0 (0.8)	0.64	0.01	0.1 [−0.5; 0.7]	1.0 [0.8; 1.4]	38.8 [29.7; 56.7]	−0.47 [−0.79;-0.08]
	post	3.0 (0.7)	3.3 (0.9)	0.15	0.10	0.3 [−0.1; 0.7]	0.7 [0.6; 1.0]	26.6 [20.6; 38.2]	0.17 [−0.24; 0.50]
Five-Point-Test									
correctly completed figures [n]	pre	28.7 (5.3)	29.0 (5.8)	0.70	0.01	0.2 [−1.3; 1.7]	2.7 [2.2; 3.7]	10.9 [8.6; 15.3]	0.78 [0.57; 0.89]
	post	30.2 (4.4)	31.3 (4.4)	0.03	0.20	0.9 [0.0; 1.7]	1.5 [1.2; 2.0]	5.4 [4.3; 7.5]	0.89 [0.79; 0.95]

### Day-to-day variability between the 2nd and 3rd day at pre and post testing

Notable differences between 2nd and 3rd day were found for the Eriksen-Flanker- (pre, reaction time, incongruent: *p* = 0.02, ɳ_p_^2^ = 0.19), Five-point test (correctly completed figures, post: *p* = 0.03, ɳ_p_^2^ = 0.20) and Stroop Color-Word test (pre, correct response, compatible: p = 0.03, ɳ_p_^2^ = 0.22) only. Coefficients of variation (CoV) for reaction times and completed figures were observed to be mainly around 5% for the Eriksen-Flanker-, Stroop Color-Word test and Five-Point test (Table 2, column 9). Digit-span testing at pre and post revealed very large COVs ranging between 11 and 39%. ICC values were mainly fair to excellent for all tests (0.40 < ICC < 0.93), except for accuracy on the Eriksen-Flanker-test (−0.47 < ICC < 0.05) and completed stages or errors on the Five-Point test (0.05 < ICC < 0.40).

### Day-to-day variability for the change score between day 1 and 2 and day 2 and 3

Change scores between pre and post testing for day 1 vs. day 2 as well as day 2 and day 3 showed insufficient relative and absolute reliability values (Table 3) ranging between 0.35 < ICC < 0.67 for the Erikson-Flanker test and −0.16 < ICC < 0.44 for the Stroop-color test. These values did not improved when comparing day 2 vs day 3. These values were even lower for the Digit-Span and Five-point test. Also absolute reliability measures showed large typical errors for the change scores during all testing conditions (Table 3).

**Table 3:** Absolute and relative reliability data of various outcomes obtained from different executive function tests before and after acute aerobic cycling exercise for day 2 and 3 comparison

				Day 1 vs. Day 2			Day 2 vs. Day 3		
	Day 1 Mean Change score	Day 2 Mean Change score	Day 3 Mean Change score	change in mean [90% CI]	SEM (Typical error) [90% CI]	ICC [90% CI]	change in mean [90% CI]	SEM (Typical error) [90% CI]	ICC [90% CI]
Erikson Flanker Test									
reaction time, compatible [ms]	−20.9	−17.4	−24.1	−0.7 [−16.8; 15.4]	29.4 [23.3; 40.3]	0.51 [0.17; 0.73]	5.3 [−9.3; 19.9]	26.7 [21.2; 36.6]	0.71 [0.46; 0.85]
correct response, compatible [%]	0.5	0.6	0.5	0.6 [−0.3; 1.5]	1.6 [1.3; 2.2]	0.42 [0.04; 0.67]	−0.6 [−1.5; 0.3]	1.6 [1.3; 2.2]	0.42 [0.04; 0.67]
reaction time, incompatible [ms]	−22.6	−22.2	−10.5	−4.5 [−19.9; 10.9]	28.2 [22.4; 38.6]	0.49 [0.14; 0.72]	10.6 [−3.6; 24.7]	25.9 [20.5; 35.4]	0.26 [−0.14; 0.57]
correct response, incompatible [%]	2.0	0.4	2.0	−1.2 [−3.0; 0.6]	3.2 [2.5; 4.4]	0.25 [−0.15; 0.56]	1.2 [−0.6; 3.0]	3.2 [2.5; 4.4]	0.25 [−0.15; 0.56]
Stroop Color Test									
reaction time, neutral [ms]	−105.2	−62.1	−56.9	22.1 [−23.3; 67.5]	83.0 [65.9; 113.8]	0.50 [0.16; 0.73]	19.9 [−20.5; 60.4]	74.0 [58.7; 101.4]	0.24 [−0.17; 0.55]
correct response, neutral [%]	−0.8	−0.2	2.5	0.3 [−1.3; 1.9]	2.9 [2.3; 4.0]	0.36 [−0.03; 0.63]	2.4 [0.8; 4.0]	2.9 [2.3; 3.9]	0.17 [−0.24; 0.50]
reaction time, incongruent [ms]	−108.5	−13.2	4.8	97.4 [−2.5; 197.3]	182.7 [145.1; 250.4]	−0.01 [−0.41; 0.36]	−6.2 [−87.1; 74.7]	148.0 [117.5; 202.8]	−0.16 [−0.55; 0.23]
correct response, incongruent [%]	1.5	−0.1	−0.6	−2.1 [−6.1; 2.0]	7.4 [5.8; 10.1]	−0.13 [−0.52; 0.26]	−0.4 [−3.6; 2.7]	5.7 [4.6; 7.9]	0.00 [−0.41; 0.36]
Digit Span									
forward [completed stages]	−0.4	0.1	−0.2	0.3 [−0.4; 1.0]	1.3 [1.0; 1.8]	0.00 [−0.41; 0.37]	−0.3 [−0.8; 0.3]	1.0 [0.8; 1.4]	0.02 [−0.39; 0.38]
forward [cumulative errors]	−0.3	0.05	−0.2	0.3 [−05; 1.0]	1.4 [1.1; 1.9]	−0.37 [−0.72; 0.02]	−0.2 [−0.8; 0.5]	1.2 [0.9; 1.6]	−0.10 [−0.50; 0.28]
backward [completed stages]	0.1	0.1	0.5	−0.1 [−0.9; 0.8]	1.6 [1.3; 2.2]	−0.63 [−0.89; −0.27]	0.5 [−0.2; 1.2]	1.2 [1.0; 1.6]	−0.18 [−0.56; 0.21]
backward [cumulative errors]	−0.4	0.1	0.3	0.5 [−0.2; 1.2]	1.4 [1.1; 1.9]	−0.39 [−0.73; 0.00]	0.2 [−0.5; 0.9]	1.3 [1.0; 1.8]	−0.30 [−0.66; 0.10]
Five-Point-Test									
correctly completed figures [n]	4.0	1.5	2.3	−1.7 [−3.1; −0.2]	2.7 [2.1; 3.7]	0.45 [0.08; 0.69]	0.7 [−1.0; 2.3]	3.0 [2.4; 4.1]	0.00 [−0.41; 0.37]
persuasive errors [n]	−0.1	−0.1	−0.2	0.1 [−0.8; 0.9]	1.6 [1.3; 2.2]	0.40 [0.02; 0.66]	−0.2 [−1.2; 0.8]	1.9 [1.5; 2.6]	0.22 [−0.18; 0.54]

All values are given with 90% confidence limits. ICC = intraclass correlation coefficient,, SEM = standard error of measurements

## Discussion

The present study assessed absolute (e.g., CoV) and relative (e.g., ICC) between-day variability of executive function (i.e., Eriksen-Flanker-test, Stroop-Color-test, Digit-Span and Five-Point-test) before and after a single bout of moderately intense aerobic cycling exercise (30 min at 70% of the heart rate reserve). The study was applied to healthy and active seniors using a three-day (habituation day, first day, second day) repeated measures design.

First, we found notable between-day habituation (from 1st to 2nd day) mainly for some time-dependent measures obtained from the Eriksen-Flanker-, Stroop-Color as well as completed figures in the Five-Point test at both pre and post testing. This is not surprising from a general viewpoint of between-day-learning. However, our testing and training session were interspersed by 7 days. Thus, also longer during between-trial breaks of up to 7 days should account for habituation effects. As these habituation effects became smaller from day 2 to day 3, relative between-day reliability (i.e., ICC) of time-dependent measures of inhibitory control (assessed with Eriksen-Flanker-test, Stroop Color-Word test) and the number of completed figures in the Five-Point test further increased from acceptable to excellent reliability. For the Stroop Color-Word and Flanker task, habituation effects have been shown previously using a short [35] or longer retest-interval [36]. The present results therefore indicate that habituation to the testing procedures should be considered in older adults, when studies aim to examine changes of inhibitory control over time. As the reliability of time-dependent measures of inhibitory control was excellent after a practice day at pre and post level, the temporal stability of these outcomes suggests that this subcomponent of executive functioning reflects stable individual differences. Despite comparatively high accuracy (>90%) on the Flanker Stroop Color-Word test, the percentage of correct responses showed remarkably lower absolute and relative reliability values than time-dependent measures in both testing scenarios. This could be due to the fact that older adults achieved very high accuracy rates, so that a ceiling effect in performance resulted in less discriminative power and variance between participants. Furthermore, a similar accuracy rate on trials assessing information processing and trials assessing inhibitory control indicates that the number of correct responses does not discriminate well between different cognitive functions and should not be used as the main outcome for the Flanker and Stroop Color-Word test. This is considered to be true, if the high accuracy rates are not solely due to participants completing the tasks with a prevention focus. This strategic inclination promotes an increase of correct responses, whereas a promotion focus reduces reaction time [37]. However, all participants were instructed to perform the task as quick and accurate as possible, so that the instructor did not systematically change the strategic inclination of the participants and a simple trade-off between reaction time and accuracy seems less likely.

Concerning working memory, the Digit-Span testing revealed poor to fair indices for both relative (ICC) and absolute (ICC) reliability, with CoV around 20% for completed stages and 40% for cumulative errors. These findings hold true for pre and post exercise testing values. For the Digit span backward, Waters and Caplan [38] have also reported a test-retest reliability that is lower than desirable in older adults [38]. However, habituation to cognitive testing increased the relative reliability of the number of completed stages. Consequently, this is the only measure of the Digit Span showing a temporal stability that justifies its use in the detection of acute or chronic effects of exercise on different aspects of executive function. However, a lower reliability of working memory measures compared to outcomes obtained from the inhibition tasks might not be test-specific. The number of trials in the Digit Span was much lower than in the Stroop Color-Word and Flanker task. It is very likely that a higher number of trials would have decreased the variability between test and retest. Therefore, increasing the number of runs on the backward and forward version of the Digit Span in addition to a habituation to the testing procedures might be most promising for the improvement of test-retest variability.

In contrast to other studies assessing the reliability of different executive function tasks, test-retest variability was measured before and after a moderate aerobic exercise session. The present study also calculated the change scores of executive function between pre and post testing. Interestingly, test-retest reliability was very similar between pre and post exercise values after habituation to the testing procedures. Thus, acute aerobic exercise does not increase the variability of executive functioning between days. Additionally, high test-retest reliability of post exercise values suggests that either the effects or the lack of effects of exercise on different aspects of executive function can adequately be reproduced. In this respect, particularly time-dependent measures of the Flanker task and the Stroop Color-Word test, completed figures in the Five-Point test and completed stages in the Digit Span backward can be used to detect acute effects of exercise on inhibitory control, task-switching and working memory, respectively.

Although widely and solely reported [39], these “relative reliability” [40] data need to be handled with caution. Intra-class correlation coefficients are highly sensitive to inter-individual variability (heterogeneity) and their magnitude can be difficult to interpret [41]. Absolute reliable data enable the comparison with other testings. Performance tests in athletes mostly require CoV levels below 5% [41] and recreational settings mostly require CoV values around or below the 10% level [18]. Higher CoV values increasingly impede a reliable detection of “real” change due to the respective intervention. However, heterogeneous populations (e.g., seniors with mental decline, chronic disease) and settings (e.g., lab, home-based) might entail meaningful baseline and exercise-induced variability of cognitive outcomes. Thus, meaningful interventional change on individual level could be “masked” by variability of the measuring and biological “system”, respectively. 10% levels of variability values are given for time-dependent variables in our group of healthy seniors. Only a minority of testing instruments (E.g., Digit-Span testing) revealed inacceptable absolute variability in seniors. Low CoV values of speed and accuracy values are needed to increase the likelihood to detected true intervention-related changes and not due to chance variations. From a scientific point of view, acute intervention studies commonly evaluate mean changes from pre- to post-testing on a group or population level with a notable and inherent amount of noise. As a consequence, reported reliability data should be used to estimate the required sample size to detect meaningful intervention effects.

The present study comprises some limitations that need to be addressed. First, we included active and healthy seniors only. It might be reasonable that participants with MCI show larger absolute variability with lower values for correct responses (ceiling effects) compared to their healthy counterparts. However, seniors per se provide large inter-individual differences in cognitive functions due to different morphological and functional aspects of brain aging (e.g., localization of lesions). Thus, our results cannot be transferred to older and frail seniors with mild to severe cognitive impairment. Moreover, day-to-day variability in post-exercise assessments might have been influenced by the participants’ dynamic capacity for adjusting cognitive processing to external demands (i.e., cognitive reserve). Second, test-retest reliability was assessed for the acute effects of exercise on executive function, so that it remains unclear whether similar ICCs can be expected in a longitudinal design. However, the reproducibility of post-exercise effects indicates that studies investigating chronic effects should control for any exercise bouts performed prior to the assessment of executive function. Third, test-retest reliability was assessed before and after a single aerobic exercise session. Consequently, the present findings do not permit any conclusions about the durability of the effects elicited by acute exercise. A review of the current literature suggests that acute benefits on executive function are maintained for at least 20 to 60 min after exercise cessation [14].

## Conclusion

Mainly time-depended variables (e.g., reaction time of the Eriksen-Flanker- and Stroop-Color test, correctly completed figures of the Five-Point test) of executive function showed notable differences at baseline and after moderate aerobic exercise between day 1 and 2. This difference decreased when comparing day 2 and 3. Thus, a notable habituation effect to the whole experimental set-up can be assumed and should be considered in future acute exercise studies on executive function. As a consequence, acute effects of moderate exercise intensity should not be overrated as the change scores are poorly reliable. Also absolute (CoV dropped from around 10% to 5% for reaction time) and relative reliability indices improved (ICC values from poor/fair to good/excellent) when comparing between-day reliability for day 2 vs. 3 compared to days 1 vs. 2. Correct responses and cumulative errors as accuracy indicators showed high percentage values indicating a ceiling effect in this population. However, reliability of accuracy turned out to be poor. Thus, highly accurate response with less variation before and after exercise can, however, cause poor reliability outcomes. Overall, Digit-span testing revealed absolute variability between 20 and 40%. This testing instrument might impede sufficient detection of exercise induced effects on executive function. Future research on baseline variability and exercise-induced effect on reliability including different types of exercise (e.g., strength, endurance, balance) in frailer and diseased seniors is needed in order to elucidate the specificity effect of exercise on executive function in the elderly population.

## Abbreviations

CI: Confidence Interval
CoV: Coefficient of Variation
HRmax: maximal Heart Rate
HRR: Heart Rate Reserve
HRrest: Resting Heart Rate
ICC: Intraclass Correlation Coefficient
MCI: Minimal Cognitive Impairment
Min: Minute
mmol/L: Millimol per Liter
ms: Millsecond
r: Pearson Correlation Coefficient
RER: Respiratory Exchange Ratio
SD: Standard Deviation
SEM: Standardized Error of Measurement
VO2peak: Peak Oxygen Uptake
